# One Step Formation of Propene from Ethene or Ethanol through Metathesis on Nickel Ion-loaded Silica

**DOI:** 10.3390/molecules16097844

**Published:** 2011-09-13

**Authors:** Masakazu Iwamoto

**Affiliations:** Chemical Resources Laboratory, Tokyo Institute of Technology, 4259-R1-5 Nagatsuta, Midori-ku, Yokohama 226-8503, Japan; Email: iwamoto@res.titech.ac.jp; Tel.: +81-45-924-5225; Fax: +81-45-924-5228

**Keywords:** ethene, ethanol, propene, metathesis, nickel, mesoporous silica

## Abstract

Increased propene production is presently one of the most significant objectives in petroleum chemistry. Especially the one-step conversion of ethene to propene (ETP reaction, 3C_2_H_4_ → 2C_3_H_6_) is the most desired process. In our efforts, nickel ion-loaded mesoporous silica could turn a new type of ETP reaction into reality. The one-step conversion of ethene was 68% and the propene selectivity was 48% in a continuous gas-flow system at 673 K and atmospheric pressure. The reactivity of lower olefins and the dependences of the ETP reaction on the contact time and the partial pressure of ethene were consistent with a reaction mechanism involving dimerization of ethene to 1-butene, isomerization of 1-butene to 2-butene, and metathesis of 2-butene and ethene to yield propene. The reaction was then expanded to an ethanol-to-propene reaction on the same catalyst, in which two possible reaction routes are suggested to form ethene from ethanol. The catalysts were characterized mainly by EXAFS and TPR techniques. The local structures of the nickel species active for the ETP reaction were very similar to that of layered nickel silicate, while those on the inert catalysts were the same as that of NiO particles.

## 1. Introduction

The mainstay of petrochemical industries in the world is still ethene (C2^=^), while the need for propene (C3^=^) is rapidly increasing due to the increasing demand of polypropene, propene oxide, *etc*. [1,2]. This trend has led to the need for the conversion of C2^=^ to C3^=^ (ETP reaction) or of increased production of C3^=^. Three kinds of measures are applied or suggested for this problem. First is the so-called mild-cracking: however, the increment of C3^=^ in this case is limited due to narrow range of applicable reaction conditions. Second is metathesis of C2^=^ and butenes (C4^=^) to form C3^=^, for instance, the ABB Lummus process [3]. Its disadvantage is the necessity for equimolar amounts of C2^=^ and C4^=^. Third is direct ETP conversion without any addition of other hydrocarbons. This would be the most desirable route, but no good catalyst for the reaction has been found so far. Supported molybdenum [4] and tungsten oxide [5] have been reported as possible catalysts, but their activity was so low as to be observed only in a closed recirculation system. On the other hand, various zeolites have been employed as catalysts for this reaction [6,7]. The reaction involves oligomerization/ polymerization of lower olefins, subsequent decomposition to yield C3^=^ or other species on the strong acid sites of the zeolites, and selective evolution of C3^=^ due to the shape-selectivity of zeolite pores. This process has the limitations of selectivity due to the shape selectivity and of catalyst lifetime owing to coke formation. The present objective is the selective formation of C3^=^ without the shape-selectivity.

The catalytic activity of Ni ion for the dimerization or oligomerization of olefins was found 50 years ago and has been widely studied [8]. In the case of heterogeneous catalysis, Ozaki *et al. *[9,10,11,12] reported the high catalytic activity of Ni/SiO_2_ for the dimerization, though severe deactivation during the reaction prevented it from being applied in the practical process. They also found that acidic supports were effective for enhancement of the catalytic activity of nickel. A similar catalytic activity was also confirmed on various Ni-zeolites [13,14] or on Ni supported on MCM-41 [15] in a closed recirculation system. Since we had already found the acidic properties of silica MCM-41 [16,17,18,19,20,21,22,23], we tried the dimerization of C2^=^ to C4^=^. During the study a subsequent reaction of the produced C4^=^ and unreacted C2^=^ to yield C3^=^ was uncovered. As a result we found that Ni ion-loaded mesoporous silica (Ni-MCM-41, abbreviated as Ni-M41) was highly active in the ETP transformation.

On the other hand the use of bio-ethanol (bEtOH) as an additive for automobile fuels has increased rapidly all over the World. This is one way of using renewable resources to suppress carbon dioxide emissions, while another challenge is the conversion of bEtOH into various olefins and their use for production of chemicals and polymers [1,2,24,25,26,27,28,29,30,31,32,33,34,35,36,37,38,39,40,41,42,43,44,45,46,47,48]. The latter would be very significant for the long-term fixation of carbon dioxide. Many efforts have therefore devoted to the development of systems for converting bEtOH to C2^=^ and other lower olefins. In particular conversion to C3^=^ is desirable due to the greater demand for C3^=^ derivatives [1,2].

Catalytic conversions of EtOH on zeolites [7,24,25,26,27,28,29,30,31,32,33,34,35] and metal oxides [36,37,38,39,40,41,42,43,44,45,46,47,48] have been widely studied. On zeolites, the activity and selectivity reported so far in many studies were insufficient. The major weakness is again catalyst deactivation [7,24,25,26,27,28,29,30,31,32,33,34,35]. EtOH can also react on metal oxide surfaces, to give various chemicals. Acid sites are widely recognized to lead to dehydration of EtOH, giving C2^=^, while basic sites lead to dehydrogenation to yield acetaldehyde (AAD) [36,37,38,39,40,41,42,43,44,45,46,47,48]. As a result, many kinds of products, for example aldehydes, ketones, C2^=^, and C4^=^, were observed on oxide catalysts. In this catalysis C4^=^ and other higher olefins were produced by oligomerization of C2^=^, but as far as we are aware, significant C3^=^ production on oxide catalysts has not been reported. The results for the ETP reaction on Ni-M41 leaded us to apply the same catalyst for the conversion of EtOH to C3^=^ since M41 is active for the dehydration of EtOH to yield C2^=^ [49,50]. This was first confirmed by us [51,52,53,54] and subsequently by Sugiyama *et al.* [55]. The pore diameters of M41 are usually 1.5–5.0 nm, and therefore the product distribution on the catalysts is not controlled by shape selectivity. The reaction mechanism/pathways are of interest, and will be suggested here. In the final part of this review the catalysts were characterized and the correlation of activities with catalyst preparation methods were also discussed.

## 2. Results and Discussion

### 2.1. Conversion of Ethene to Propene on Ni-M41 Catalysts

The reactions on Ni-M41 were examined as a function of reaction temperature. The dimerization of C2^=^ to C4^=^ mainly proceeded at 573 K. When 0.5 g of Ni-M41(Si/Ni=15) was used, the degrees of conversion of C2^=^ and the selectivity to C4^=^ reached 43 and 93%, respectively. The production ratio of 1-, *trans*-2-, and *cis*-2-butene was 0.5:1.0:0.3. At 673–723 K the major products were C3^=^ and C4^=^. The respective conversion levels were dependent on the partial pressure of C2^=^ and the contact time, as shown later. Hexenes, the product of C2^=^ trimerization, were observed at the wide temperature range but the yields were always less than 5%.

When silica gel was used as the support instead of M41 and nickel ion was loaded with the usual impregnation method, both the conversion level and the selectivity of C3^=^ were very poor. In addition, no C4^=^ was produced on M41 alone, indicating the necessity of nickel ion for the reaction. It follows that the coexistence of nickel ion and mesoporous structure of the support make the C3^=^ formation possible. The catalyst was continuously used at 673 K for 10 h to determine the possible deactivation. Small changes in the catalytic activity for the formation of C3^=^ were observed in the initial stage, but the activity became stable within 2 h and no deactivation was found during the 10 h experiment. The XRD patterns and the surface areas of Ni-M41 remained unchanged after the catalytic runs. Thus the stability of the present Ni-M41 catalysts under the present reaction conditions could be confirmed. 

The correlations between the product distribution and the reaction conditions were then investigated. In the range P_C_2_H_4__=10–50%, the conversion levels of C2^=^ and to C3^=^ and C4^=^ increased monotonously with increasing P_C_2_H_4__. At P_C_2_H_4__=49.7%, the respective conversions to C3, C4, and C6 olefins were 33, 29, and 6% on 0.3 g of Ni-M41(20). The carbon balance was 99.8% in each experiment, which indicates almost no production of “unknown products”. The degree of conversion to C3^=^, 33%, appears to rather small but it should be noted that the concentration of unreacted C2^=^ was about 34% under the present conditions and the ratio of C3^=^/C2^=^=33/34 in carbon basis would be sufficiently great. 

**Figure 1:**
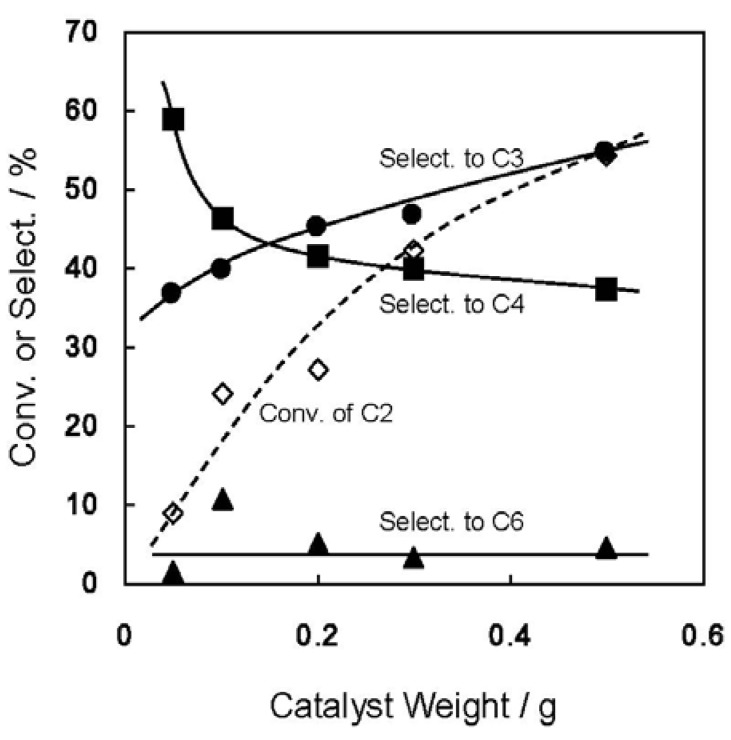
Change in ethene conversion and product distribution at 673 K with weight of Ni-M41(43). The codes C2–C6 mean ethene, propene, butenes, and hexenes.

Figure 1 shows the change in product distribution as a function of the weight of Ni-M41 employed, *i.e.*, the contact time dependence of the reaction. Clearly, longer contact times resulted in greater conversion of C2^=^ and better selectivity for C3^=^, while the selectivity for C4^=^ decreased and that of hexenes was almost constant. Propene is indeed the secondary product in the consecutive reaction of C2^=^ on Ni-M41. At 0.5 g of Ni-M41(43), the degrees of C2^=^ conversion and C3^=^ selectivity were 55 and 54%, respectively.

**Figure 2:**
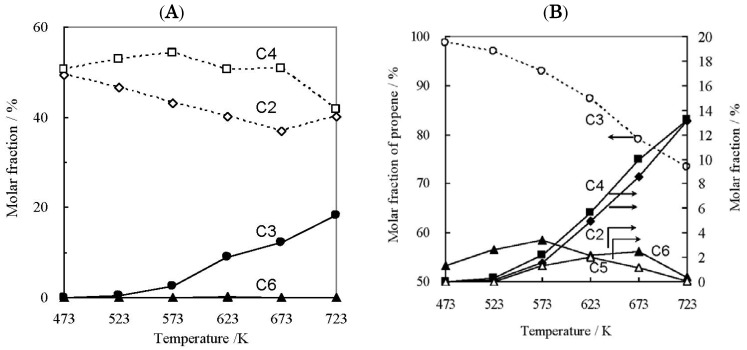
Metathesis reaction of ethene and 1-butene (**A**, P_C_2_H_4__ = P_C_4_H_8__ = 5%) or propene (**B**, P_C_3_H_6__ = 10%) on 0.3 g of Ni-M41(15). The codes C2–C6 mean ethene, propene, butenes, pentenes, and hexenes. In Figure 2B, the left vertical axis is the amount of propene and the right those of the products.

The reaction of C2^=^ and 1-butene was then studied to clarify the mechanism of the C3^=^ formation and the results are summarized in Figure 2A. One can recognize the selective formation of C3^=^ on Ni-M41 at the temperature of 623 K and higher. The increment in the amount of C4^=^ at 523–573 K is due to the dimerization of C2^=^. The selective production of C3^=^ would indicate the progress of the metathesis reaction on this catalytic system. To confirm the reaction pathway in more detail, we examined two kinds of reactions. The first was the reaction of 1-hexene. When 1-hexene was introduced onto the Ni-M41 catalyst, methane, C2^=^, C4^=^, and pentenes were produced, besides C3^=^, indicating the random scission of carbon-carbon bonds of 1-hexene. This indicates little possibility that C2^=^ and 1-butene first afford hexenes and the resulting hexenes homolytically decompose to give C3^=^ selectively in the experiments of Figure 2A. The second reaction examined was the retro-metathesis reaction. Namely the reaction of C3^=^ on Ni-M41 was investigated and shown to readily proceeded to yield equimolar C2^=^ and C4^=^ as shown in Figure 2B. The amounts of by-products were always small. It was further confirmed in separate experiments that the parent M41 was not active for the reaction of C2^=^ and C4^=^. All of the results therefore strongly suggest the metathesis reaction on Ni-M41 and that the active center for the catalysis would be nickel ion. 

**Figure 3:**
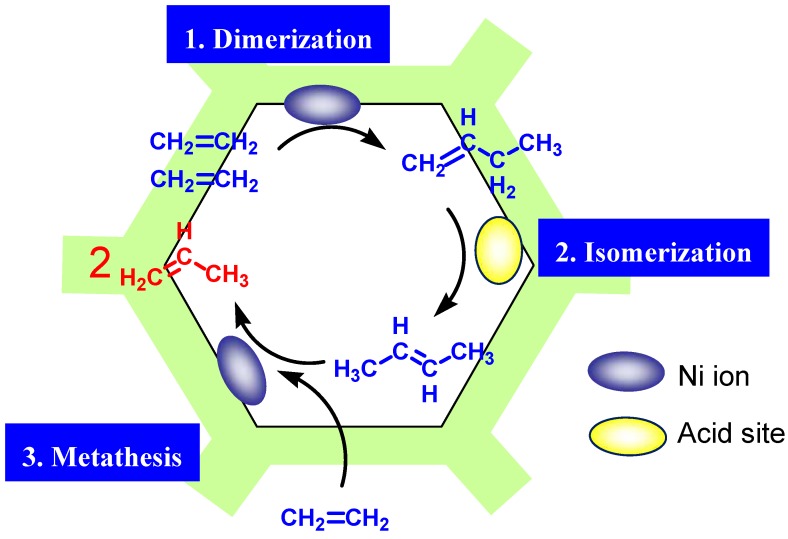
Proposed reaction mechanism for the conversion of ethene to propene on Nickel ion-loaded MCM-41.

Although at present we cannot preclude the possibility of a decomposition mechanism of higher olefins because other types of reaction mechanisms have been suggested on Cr [56] or Zr [57], we believe that the metathesis mechanism (Figure 3) is the most plausible reaction mechanism for the C3^=^ formation on Ni-M41. That is, at first two C2^=^ molecules dimerize to give 1-butene on Ni, and the resulting 1-butene then isomerizes to 2-butene on the acid sites of M41, and finally the metathesis of the produced 2-butene with unreacted C2^=^ proceeds to form C3^=^ on Ni. The acidic properties of M41 silica were already been reported by us [16,17,18,19,20] and the other research groups [15,21,22,23] and the isomerization of 1-butene to 2-C4^=^, a typical acid-catalyzed reaction, was indeed confirmed on silica M41 [15].

As has been summarized by Grubbs [58,59] and Arpe [1], the metathesis reaction is one of the most important organic reactions. Despite world-wide study it is well known that the catalytically active species for the reaction are confined to Mo, W, Ru, and Re. The present results might suggest that nickel-ion loaded mesoporous silica is also active for the metathesis of C4^=^ and C2^=^ to yield C3^=^ in the gas-phase flow reaction. Mori *et al.* [60] suggested the possibility of metathesis on a Ni(0) complex in their discussion, while Baker *et al.* [61] concluded no progress of a metathesis reaction on Ni complexes. At the moment, no reports claim nickel ion as a catalytically active species for metathesis. It is noteworthy that the surface density of Ni is approximately 0.5 Ni/nm^2^ in the case of Ni-M41(20) on the assumption of the even distribution of nickel on the surface. The valence of nickel ion in the mesoporous silica were not studied here. There are two possibilities for the redox cycles of nickel species, Ni(I)-Ni(III) and Ni(0)-Ni(II). In Section 3.3 the TPR experiments will indicate the difficult reduction of nickel species loaded on M41 to Ni(0), which would be one important factor for generation of the catalytic activity. Therefore we speculate that the Ni(I)-Ni(III) system would be the possible redox cycle for the metathesis reaction. The stability of Ni(I) in the zeolites [62,63,64] and mesoporous materials [65,66] support the speculation that Ni(I) is an active center and a Ni(III) carbene is produced as an intermediate. 

Finally the effectiveness of other metal ions for this reaction is briefly introduced here. The conversion levels of C2^=^ on Al (22), Ti (30), V (22), Cr (43), Mn (20), Fe (25), Co (16), Cu (37), Zn (28), Zr (23), Mo (30), or W (30) loaded M41 were all 5% or less at 673 K, and most of the products were “unknown products”. It should be noted, however, that the gas-phase dimerization-isomerization-metathesis of C_2_^=^ on tungsten catalysts was independently reported by Basset *et al.* [67] and the others [68]. The difference clearly results from the discrepancy of reaction conditions. Ru or Re loaded MCM-41 were prepared separately through the conventional impregnation method and employed as the catalyst for the present reaction at 673 K because of its high activity reported at lower temperatures, but no activity for the 3C_2_^=^→ 2C_3_^=^ reaction was observed in our experiments. This would be due to the lack of activity of Ru or Re for the dimerization of C_2_^=^ and the difference of the reaction temperature applied. Clearly only nickel ion shows the unique activity for the ETP conversion in the gas-phase reaction at 673 K. The reason for the specific activity of nickel ion on MCM-41 would be a target of the future work.

### 2.2. Reaction of Ethanol on Ni-MCM-41

**Figure 4:**
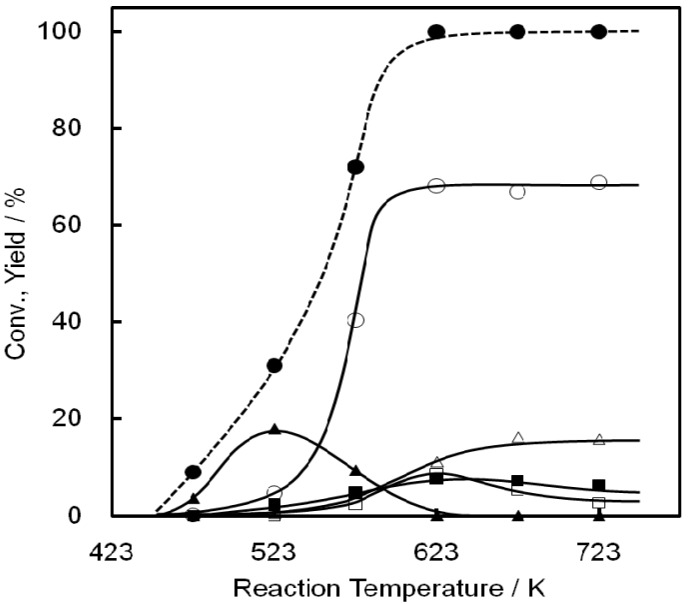
Reaction temperature dependence of conversion of EtOH on a Ni-M41(23) catalyst. Catalyst wt. 0.2 g, Flow rate 10 mL/min, P_EtOH_ 5.6 kPa (N_2_ balance). Conversion of EtOH (closed circle), yield of C2^=^ (open circle), C3^=^ (open triangle), C4^=^ (open square), DEE (closed triangle), and AAD (closed square).

The influence of temperature on EtOH conversion over Ni-M41 is summarized in Figure 4. Many kinds of products were formed in addition to C2^=^. Diethyl ether (DEE) was mainly obtained at around 523 K. DEE has been reported earlier as an intermediate compound in the dehydration, decomposing to yield EtOH and C2^=^ at higher temperatures [49,50]. The C2^=^ yield increased sharply at 573 K, and reached ca. 70% at 623 K or above. The C4^=^ yield reached a maximum at 623 K, while maxima in C3^=^ yield occurred at 673 and 723 K. Notably, AAD was formed at 573–723 K, although not in large amounts, which will be discussed later.

The stability of Ni-M41 was examined at 673 K. The catalytic activity did not change during 20 h of continuous time on stream. In addition, the carbon-based mass balances were always ca. 100%, within the experimental errors. The results demonstrate the stable catalytic activity of Ni-M41. However, there is the possibility that losses of catalytic activity could not be determined under these conditions because the catalytic activity of Ni-M41 was very high, as will be revealed in a following paragraph, and the conversion levels of EtOH were always ca. 100%. The yields of C2^=^, C3^=^, C4^=^, and AAD were 67, 16, 5, and 7 %, respectively. The values should be compared with those of the reaction of C2^=^ on the same catalyst reported previously (see Section 3.1). At 673 K and *P*_C2=_=10 vol %, the C2^=^ conversion and selectivity to C3^=^ and C4^=^ were reported to be 42, 47, and 40 %, respectively [49,50]. Clearly, the product distribution for the EtOH reaction is different from that of the C2^=^ reaction. This difference might result from a change in active sites through adsorption of EtOH, AAD, or intermediates, or from a difference in reaction mechanism. The dependence on the partial pressure of EtOH was also studied at *P*_EtOH_ = 3.0–13.2 kPa and a space velocity (SV) of 1,000 h^−1^. Little dependence of C3^=^ formation on *P*_EtOH_ was observed under the present conditions, probably indicating strong adsorption of EtOH on the active sites.

A similar reaction of EtOH on proton-exchanged ZSM-5 zeolites was reported to be retarded in the presence of water vapor [7,24,25,26,27,28,29,35], so the effect of water addition on the catalytic activity of Ni-M41 was studied here. When EtOH/water ratios were varied in the range 100:0–75:25 (*w*/*w*), the EtOH conversion levels and product distribution changed only a little. This result is very significant for the application of the present system to bEtOH conversion, because coarsely distilled bEtOH usually contains 5–10 vol % water.

**Figure 5:**
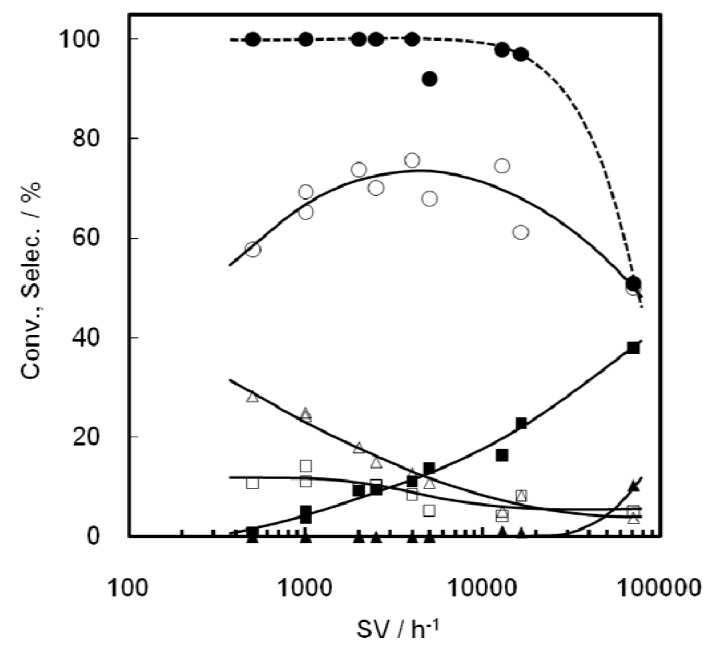
Conversion of EtOH on Ni-M41(23–28) as a function of space velocity. Catalyst wt. 0.05–0.4 g, Flow rate 10–300 mL/min, P_EtOH_ 5.6 kPa (N_2_ balance). Conversion of EtOH (closed circle), selectivity of C2^=^ (open circle), C3^=^ (open triangle), C4^=^ (open square), DEE (closed triangle), and AAD (closed square).

The product distribution as a function of space velocity was studied on Ni-M41 at 673 K. The dependence is summarized in Figure 5. At SV = 70,000 h^−1^ the conversion level of EtOH was ca. 50%, but at 20,000 or below it increased to 95% or more. It follows that Ni-M41 is very active for the catalytic conversion of EtOH. 

The product distribution depends strongly on the space velocity. At higher SVs (in the 10,000–100,000 h^−1^ region) AAD is produced in large amounts, and the amount decreases monotonically with decreasing SV. DEE was also produced and its formation showed a behavior similar to that of AAD, although the amount formed was very small. C2^=^ was always a major product in the reaction, and its yield reached a maximum at 2,000–3,000 h^−1^. On the other hand the yields of C3^=^ and C4^=^ gradually increased with decreasing SV, indicating that these compounds are products from the terminal phases of consecutive reactions. On the basis of the above results, we suggest the following reaction pathways to form C3^=^ *via* DEE and C2^=^ as intermediates:

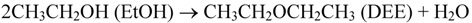
(1)

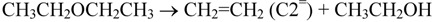
(2)

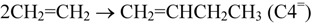
(3)

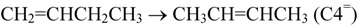
(4)

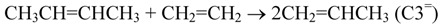
(5)

**Figure 6:**
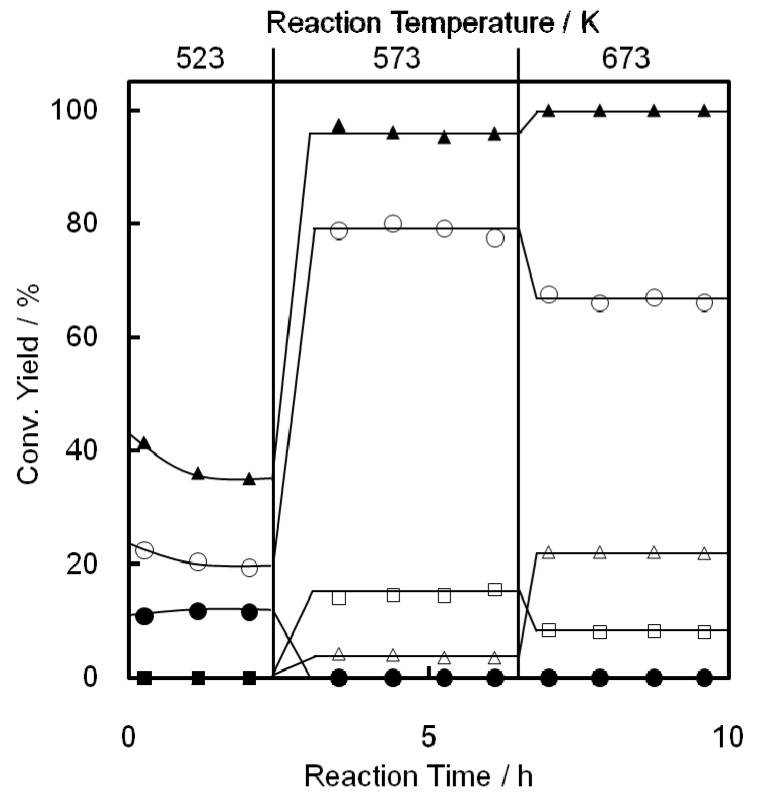
Change in conversion of DEE on Ni-M41(23) with reaction temperature and time. Catalyst wt. 0.2 g, Flow rate 10 mL/min, P_DEE_ 5.1 kPa (N_2_ balance). Conversion of DEE (closed triangle), yield of C2^=^ (open circle), C3^=^ (open triangle), C4^=^ (open square), EtOH (closed circle), and AAD (closed square).

Although the formation of DEE from EtOH and the subsequent decomposition to yield C2^=^ and EtOH on a M41 catalyst was already reported [49], the progress was here confirmed this separately. In Figure 6 DEE was employed as a substrate and the product distribution was examined as a function of reaction temperature. At 523 K the conversion of DEE to EtOH and C2^=^ was again confirmed. At 573 K the major products were C2^=^ and C4^=^ and a small amount of C3^=^ was produced. At 673 K the produced C2^=^ and C4^=^ were converted to C3^=^ through metathesis, as reported previously [49,50].

There was one more important question in the results of Figure 5: the meaning of the increment in AAD at higher SV values. The results showed that AAD was an intermediate to form C2^=^, although the reaction was never suggested. The following reaction is widely accepted to proceed on various catalysts:

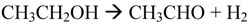
(6)

The possibility that mixtures of AAD and hydrogen really give C2^=^ is examined here. An equimolar mixture of AAD and H_2_ (6 kPa, respectively, N_2_ balance) was introduced onto the Ni-M41 catalyst at 573–773 K and a SV of 4,600 h^−1^, but no reaction except for condensation of AAD and low levels of methane and C2^=^ formation (2–3%, respectively) was observed. The possibility that the reverse reaction of Equation (6) and subsequent dehydration of resultant EtOH might lead to the formation of C2^=^ is therefore ruled out. Next, an equimolar mixture of EtOH and AAD (5 kPa respectively, N_2_ balance) was introduced onto the Ni-M41 catalyst at 673 K and a SV of 990 h^−1^. The conversion levels of EtOH and AAD were 100 and 65% and the selectivities to C2^=^, C3^=^, and C4^=^ were 61, 15, and 12%, respectively. The selectivity was very similar to that observed in Figure 4 and Figure 5. AAD can be converted into C2^=^ in the presence of EtOH and Equations (3–5) subsequently proceed in this reaction system.

In the experiments of Figure 5 trace amounts of ethyl acetate (ETA) were observed, although the amount was not quantified. The following reactions could be suggested for the formation of C2^=^ from AAD and EtOH:

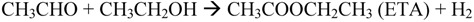
(7)

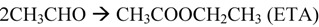
(7’)

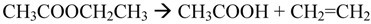
(8)

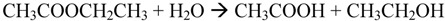
(8’)

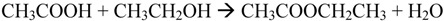
(9)

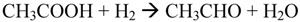
(10)


Equation (7’) is well-known as the Tishchenko reaction, and Equation (9) as the Fisher Esterification. The experimental results indicate the progress in Equation (7) instead of (7’) on Ni-M41. It is already known that hydrolysis of ETA [Equation (8’), the reverse reaction of Equation (9)] gives acetic acid and EtOH, but Equation (8) is not popular. The reverse reaction of Equation (8), however, was already confirmed to proceed catalytically and was put into practical use by Showa Denko K.K., Japan [69]. To postulate Equation (8) is therefore legitimate. The sequence of reactions (6)–(7)–(8)–(9)–(8) would result in the formation of C2^=^ from AAD and EtOH through ETA and acetic acid as the intermediates.

### 2.3. Characterization of Nickel Species Loaded on the Mesoporous Silica

Three kinds of Ni-loaded M41 samples were prepared to clarify the state of the nickel ion. They were prepared by TIE, impregnation (IMP), equilibrium adsorption (EA) of [Ni(NH_3_)_x_]^2+^ as shown later. The colors of the EA, TIE and IMP catalysts were pale ivory, pale ivory and pale blackish purple, respectively. The following results and discussion will be described on the premise of no essential difference in the pore structures among the M41 samples employed here. 

**Figure 7:**
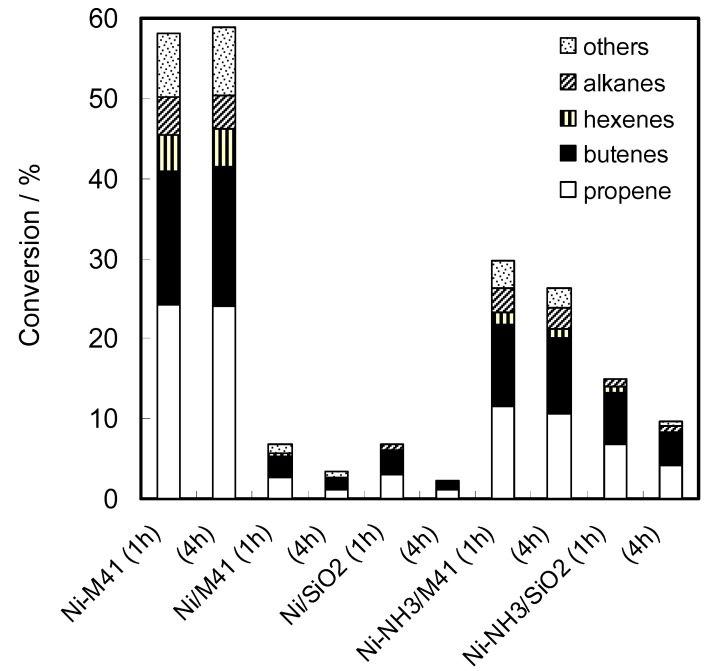
Catalytic activity of nickel-ion loaded catalysts for the ETP reaction at 673 K. The reaction times after the beginning of the reaction are described in the parentheses. Catalysts: Ni-M41, Ni/M41, Ni/SiO_2_, Ni-NH_3_/M41, and Ni-NH_3_/SiO_2_. Catalyst 0.3 g, total flow rate 11 mL/min, 0.1 MPa, ethene 9.3% and water 1.4% (N_2_ balance). Alkanes: methane and ethane.

The activity of the TIE catalyst for the ETP reaction was first compared with those of the IMP catalysts. Figure 7 shows the catalytic activities of Ni-M41, Ni/M41, and Ni/SiO_2_ at 1 and 4 h after the beginning of the reaction. Only the Ni-M41 catalyst prepared by the TIE method showed high and stable activity for the ETP reaction, while the activity of Ni/M41 or Ni/SiO_2_ was very low and decreased with the reaction time. To clarify the origin of the great difference between the activities of TIE- and IMP-catalysts, the catalysts were characterized by various methods. Surface areas of Ni-M41 and Ni/M41 calcined at 773 K were 856 and 822 m^2^/g, respectively. The values indicate little correlation between the surface area and the catalysis. The XRD measurements did not confirm any nickel-related crystalline phases on the TIE-catalysts, but showed the formation of NiO particles on the IMP catalysts. 

**Figure 8:**
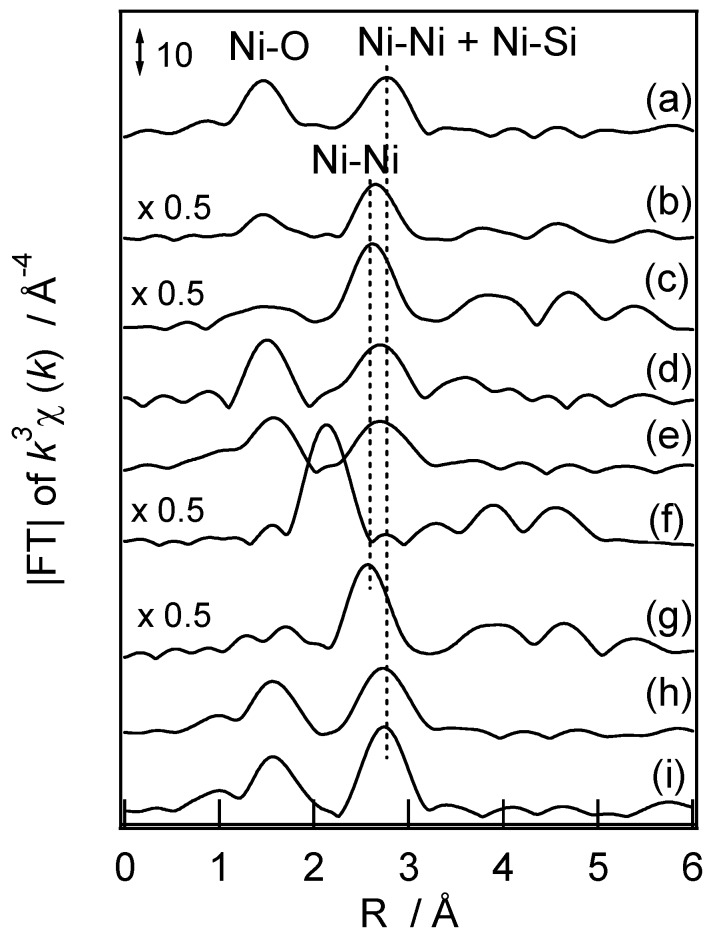
Fourier transforms of *k*^3^-weighted EXAFS spectra of (**a**) Ni-M41; (**b**) Ni/M41; (**c**) Ni/SiO_2_; (**d**) Ni-NH_3_/M41; **(e**) Ni-NH_3_/SiO_2_; (**f**) Ni foil; (**g**) NiO; (**h**) Ni-silicate (antigorite); and (**i**) Ni-silicate (talcite). Δ*k*: 3.2–12 Å^−1^.

More detailed characterization of the supported nickel species has been carried out by using the EXAFS and TPR techniques. Figure 8 shows radial structure functions (RSFs) of Ni-ion loaded catalysts and reference compounds. Most of the samples except the Ni foil gave two peaks at 0.15–0.16 and 0.26–0.28 nm though their respective intensities were depended on the samples. The latter peaks indicate the presence of Ni-Ni pairs. The conventional curve fitting analysis was applied to the spectra to determine the interatomic distance and the coordination numbers around the nickel atom and the results are summarized in Table 1. It should be noted in the table that the accuracy of coordination numbers estimated for the second coordination sphere has some uncertainty because the range of EXAFS spectra adopted here was limited to 120 nm^−1^. We employed Ni foil, NiO, and two kinds of layered nickel silicates as the reference compounds. 

**Table 1:** XAFS parameters of Ni ion in various Ni-loaded silica catalysts.

Sample	Shell	C. N. ^a^	D/nm ^b^	Δσ^2^/nm^2^ ^c^	R/% ^d^
Ni-M41	Ni-O	6.9	0.208	6.08 × 10^−^^5^	16.2
	Ni-Ni	5.1	0.305	4.90 × 10^−^^5^	4.4
	Ni-Si	2.0	0.336	1.02 × 10^−^^5^	
Ni/M41	Ni-O	^f^			
	Ni-Ni	10.3	0.300	5.04 × 10^−^^5^	3.0
Ni/SiO_2_	Ni-O	^f^			
	Ni-Ni	11.6	0.296	3.84 × 10^−^^5^	1.3
Ni-NH_3_/M41	Ni-O	^f^			
	Ni-Ni	3.8	0.305	3.25 × 10^−^^5^	6.1
	Ni-Si	2.4	0.337	0.6 × 10^−^^5^	
NiO	Ni-O	6	0.208		
	Ni-Ni	12	0.295		
Ni-talcite ^e^	Ni-Ni	6.0	0.305		
	Ni-Si	5.0	0.327		
Nepouite ^e^	Ni-Ni	6.0	0.309		
	Ni-Si	2.4	0.327		

^a^ Coordination number; ^b^ Interatomic distance; ^c^ Debye Waller factor; ^d^ Agreement factor; ^e^ Cited from reference [70]; ^f^ No appropriate fits could be obtained.

Table 1 and Figure 8 reveal several important points from the comparison with the literature [70,71,72,73,74,75,76,77,78,79,80]. The distance and the coordination number of the first shells (oxygen backscatterer) on Ni-M41 indicate the presence of hexacoordinated Ni^2+^ 6c ions [71] in the TIE sample. Yang *et al.* [72] reported that the nickel ion substituted for Si ion in the M41 framework has a tetrahedral coordination structure, which indicates a complete difference between the coordination states of the nickel ions in the present TIE sample and in the Ni-MCM-41 prepared by the sol-gel method. The distances of the second shell (Ni and Si backscatterer) of Ni-M41, 0.305 and 0.336 nm, were longer than those of NiO and Ni/M41, and similar to those of layered nickel silicates. The findings concerning the first and second shells strongly indicate that the nickel ion in the TIE sample has a layered nickel silicate-like structure. The typical layered nickel silicates consist of a NiO_6_ layer sandwiched by one or two silica layers [70,73,74,81,82]. The EXAFS spectra of the two types of nickel silicates [Table 1 and Figures 8h,i], however, were quite similar to each other, as has already been reported by several workers [70,71,75]. At the moment therefore we cannot determine the exact surface structure of nickel ion on the basis of the EXAFS spectra. In contrast, the Ni/M41 and Ni/SiO_2_ catalysts gave spectra assignable to NiO species because the Ni-Ni distance was shorter than those of Ni-M41 and the layered-nickel silicates. It follows that the preparation methods have an essential effect for the appearance of the catalytic activity through the change in loading states of nickel ion on the supports. The role of layered-nickel silicates on M41 for the catalysis will be described in the later paragraphs in more detail.

The nickel species in the TIE catalyst gave a reduction peak at 931 K in the TPR experiments. This temperature was much higher than those of Ni/M41 (839 K), Ni/SiO_2_ (746 K), and NiO alone (673 K). In TPR profiles of the nickel silicates, similar to previous works [74,76], the broad reduction peaks were observed on these samples. On the basis of many TPR experiments reported so far [72,79,83,84], we can summarize the TPR peak regions of nickel on silica as follows: Ni-oxide, the cationic form of nickel on the silica surface, and the nickel ion forming some surface composite compounds could be reduced at ca. 600–800, 800–900, and 900–1,000 K, respectively. The reduction temperature of Ni-M41 clearly falls within the region of the reduction of composite compounds. This further supports the above conclusion that the nickel ion in the TIE sample might form the layered nickel silicate like-structure on the surface. 

The amounts of H_2_ consumed in the TPR experiments of the TIE sample and the layered silicates were almost equal to those of nickel ion contained in the respective samples. In contrast, the IMP catalysts gave a much higher ratio than unity. The composition of Ni oxide prepared by thermal decomposition of nickel carbonate at 773 K was reported to be NiO_1.13_ and its color was black [85]. In addition, Ni-oxide prepared by the impregnation onto silica support was suggested to be most probable Ni_2_O_3_ species [84]. The larger TPR peaks than those expected from H_2_/Ni = 1 and the pale blackish purple color of the present IMP catalysts both indicate the existence of the mixture of Ni_2_O_3_ and NiO on the silica surface. 

The surface layered nickel silicate is reported to be produced by loading of nickel ion as amine-complexes onto silica in a basic aqueous solution and then heating them in air at 623–1,073 K [71,72,76,78,79,80,83,86,87]. Hadjiivanov *et al.* reported that the EA of [Ni(NH_3_)_x_]^2+^ onto silica gel at pH 12.3 and the subsequent calcination at 623 K is effective for its preparation with ease [83]. We have here applied their method to prepare the samples containing the surface layered nickel silicate (the EA catalysts) to evaluate its role for the catalysis, in which the EA samples were finally calcined at 773 K. The XRD patterns of the Ni-NH_3_/M41 and Ni-NH_3_/SiO_2_ catalysts did not show any diffraction peaks assignable to the layered nickel silicate, indicating the domain size of the surface layered nickel silicate was not large. The fine structure of nickel ion in the EA catalyst was studied by XAFS and the results are shown in Figure 8 and Table 1. It is clear that the spectra were very similar to those of the layered nickel silicate. The TPR profiles of the EA catalysts were separately measured. They have much resemblance to that of Ni-M41 though the reduction temperatures, 898 and 927 K, were somewhat lower than that of Ni-M41. All of the results clearly indicate the formation of the layered nickel silicate on the silica surface by the EA method, as has been reported by several authors. 

The catalytic activity of the Ni-NH_3_/M41 and Ni-NH_3_/SiO_2_ samples is shown in Figure 7. It was lower than that of the Ni-M41 sample (the TIE catalyst) while much greater than those of the IMP samples. Deactivation during the reaction was also observed with the EA samples, but the degrees were smaller than those of the IMP catalysts. More detailed investigation into the preparation conditions of the EA catalysts possibly leads to raising their catalytic activity to the same levels as that of the TIE catalyst. This estimation was indeed realized partly by Lehman *et al.* [88] All results presented here showed that the TIE method is the most effective to prepare the active nickel ion-loaded catalysts and the high catalytic activity would result from the effective formation of surface layered nickel silicate-like structure. It would be worth to note that we attempted to measure the dispersion of nickel metal on the TIE samples after the TPR experiments by the conventional CO adsorption [89,90] but we could not find any irreversible adsorption of CO. This means that the state of nickel metal on the TIE catalysts is entirely different from those on the conventional catalysts, which would be a target for the future study.

## 3. Experimental Section

M41 was prepared in the reported procedure [91,92,93] using C_12_H_25_N(CH_3_)_3_Br as the template and colloidal silica as the silica source. Nickel ion was loaded onto M41 by the template ion exchange (TIE) method using an aqueous nickel nitrate solution [49,50,91,92,93], the conventional impregnation method, or the equilibrium adsorption method, as will be summarized in Section 3.3. The samples were named as Ni-M41, Ni/M41, and Ni-NH_3_/M41, respectively. As-prepared Ni-loaded MCM-41 was calcined at 773 K for 6 h in air, in which the sample was thinly (less than 2 mm thick) spread onto a ceramic board and heated at 0.2–0.5 K a minute. The slow heating with the shallow bed method was important to obtain good and reproducible catalytic activity. The Brunauer-Emmett-Teller (BET) surface area and the Barrett-Joyner-Halenda (BJH) pore diameter determined by a N_2_ adsorption measurement were 873–1010 m^2^g^−1^ and 2.2 nm, respectively. The hexagonal structure of the resulting M41 was confirmed by the appearance of 2*θ *= 2.580°, 4.476°, and 5.124° peaks in the X-ray diffraction patterns (Cu K_α_, Ni filter), which corresponded to (100), (110), and (200), respectively. The Si/Ni atomic ratios in the calcined samples were 23–28 unless otherwise stated (values were shown between brackets in the sample names). The Si/Al atomic ratios were 237–243, in which the origin of Al was an impurity of the colloidal silica raw material. The catalytic reaction was carried out using a fixed-bed flow reactor at atmospheric pressure. The catalyst (0.05–0.5 g) was loaded in the reactor, heated in N_2_ at 673 K, and then C2^=^ or EtOH (P_C2=_ or *P*_EtOH_=2.8–12.8kPa, N_2_ balance, total flow rate 10–300 mL min^−1^) was let into the reactor at a desired temperature with a mass flow controller or a syringe-type microfeeder. The product distribution was determined by an on-line gas chromatograph and the yields and selectivity were calculated on the carbon basis.

## 4. Conclusions

Our reports have for the first time claimed the gas-phase metathesis on nickel-containing catalysts at around 673 K. The specific characteristics of this finding are nickel, gas-phase, and high temperature. The reaction mechanism is suggested to be the dimerization of C2^=^, the isomerization of the produced 1-C4^=^, and the metathesis of C4^=^ and C2^=^ to yield C3^=^. The reaction was then expanded to ethanol and we could also get C3^=^ from EtOH. Two reaction routes for the formation of C2^=^ from EtOH on Ni-M41 were revealed and proceeded in parallel. One is the dehydration route *via* DEE as intermediate. The other is a complicated route through AAD and ETA as intermediates. The reaction rate of the latter route is slower than that of the former, since the formation of AAD was observed in a wide range of SV values. The C2^=^ produced was converted to C3^=^ through dimerization, isomerization, and metathesis. The present results indicate that the formation of C3^=^ from C2^=^ or EtOH could be achieved by not using the shape selectivity well known in zeolite catalysis. The layered nickel-silicate like structure would be the active species for the new type of ETP reaction. More detailed investigation of the present system would develop a new horizon in gas-phase metathesis.

## References

[1] Arpe H.J. (2003). Industrial Organic Chemistry.

[2] Olah G.A., Molnar A. (2003). Hydrocarbon Chemistry.

[3] Banks R.L., Kukes S.G. (1985). New developments and concepts in enhancing activities of heterogeneous metathesis catalysts. J. Mol. Catal..

[4] O’Nill P.P., Rooney J.J. (1972). Direct transformation of ethylene to propylene on an olefin metathesis catalyst. J. Am. Chem. Soc..

[5] Yamaguchi T., Tanaka Y., Tanabe K. (1980). Isomerization and disproportionation of olefins over tungsten oxides supported on various oxides. J. Catal..

[6] Lu J., Zhao Z., Xu C., Duan A., Zhang P. (2006). CrHZSM-5 Zeolites - Highly Efficient Catalysts for Catalytic Cracking of Isobutane to Produce Light Olefins. Catal. Lett..

[7] Oikawa H., Shibata Y., Baba T. (2006). Highly selective conversion of ethene to propene over SAPO-34 as a solid acid catalyst. Appl. Catal. A Gen..

[8] Speiser F., Braunstein P., Saussine L. (2005). Catalytic Ethylene Dimerization and Oligomerization: Recent Developments with Nickel Complexes Containing P,N-Chelating Ligands. Acc. Chem. Res..

[9] Shiba T., Ozaki A. (1953). A catalyst consisting of nickel oxide and silica. I. The catalytic activity of ethylene polymerization caused by vacuum heating. Nippon Kagaku Zasshi.

[10] Ozaki A. (1956). Mixed catalyst composed of nickel oxide and silica. V. Relation between the composition and the activity for the polymerization of ethylene. Nippon Kagaku Zasshi.

[11] Kimura K., Ai H., Ozaki A. (1970). Tracer study of ethylene dimerization over nickel oxide-silica catalyst. J. Catal..

[12] Sohn J.R., Ozaki A. (1980). Acidity of nickel silicate and its bearing on the catalytic activity for ethylene dimerization and butene isomerization. J. Catal..

[13] Ghosh A.K., Kevan L. (1990). Electron spin resonance studies of ethylene dimerization catalyzed by nickel species on Y zeolites. J. Phys. Chem..

[14] Zheng L., Wang G., Bai X. (1986). Interaction of nickel ions with ethylene molecules in ethylene dimerization over Ni-X zeolites. Stud. Surf. Sci. Catal..

[15] Hertmann M., Poppl A., Kevan L. (1996). Ethylene Dimerization and Butene Isomerization in Nickel-Containing MCM-41 and AlMCM-41 Mesoporous Molecular Sieves: An Electron Spin Resonance and Gas Chromatography Study. J. Phys. Chem..

[16] Iwamoto M., Tanaka Y., Sawamura N., Namba S. (2003). Remarkable Effect of Pore Size on the Catalytic Activity of Mesoporous Silica for the Acetalization of Cyclohexanone with Methanol. J. Am. Chem. Soc..

[17] Tanaka Y., Sawamura N., Iwamoto M. (1998). Highly effective acetalization of aldehydes and ketones with methanol on siliceous mesoporous material. Tetrahedron Lett..

[18] Ishitani H., Iwamoto M. (2003). Selective aldol reactions of acetals on mesoporous silica catalyst. Tetrahedron Lett..

[19] Murata H., Ishitani H., Iwamoto M. (2010). Synthesis of Biginelli dihydropyrimidinone derivatives with various substituents on aluminium-planted mesoporous silica catalyst. Org. Biomol. Chem..

[20] Murata H., Ishitani H., Iwamoto M. (2010). Highly ordered aluminum-planted mesoporous silica as active catalyst for Biginelli reaction and formyl C-H insertion reaction with diazo ester. Phys. Chem. Chem. Phys..

[21] Trong D., Joshi P.N., Lemay G., Kaliaguine S. (1995). Acidity and structural state of boron in mesoporous boron silicate MCM-41. Stud. Surf. Sci. Catal..

[22] Yamamoto T., Tanaka T., Funabiki T., Yoshida S. (1998). Acidic Property of FSM-16. J. Phys. Chem. B.

[23] Ito A., Kodama T., Maeda S., Masaki Y. (1998). Selective acceleration for deprotection of benzyl ethers with Ti-HMS. Tetrahedron Lett..

[24] Phillips C.B., Datta R. (1997). Production of Ethylene from Hydrous Ethanol on H-ZSM-5 under Mild Conditions. Ind. Eng. Chem. Res..

[25] Brando P., Philippou A., Rocha J., Anderson M.W. (2002). Dehydration of Alcohols by Microporous Niobium Silicate AM-11. Catal. Lett..

[26] Aguayo A.T., Gayubo A.G., Atutxa A., Olazar M., Bilbao J. (2002). Catalyst Deactivation by Coke in the Transformation of Aqueous Ethanol into Hydrocarbons. Kinetic Modeling and Acidity Deterioration of the Catalyst. Ind. Eng. Chem. Res..

[27] Aguayo A.T., Gayubo A.G., Tarro A.M., Atutxa A., Bilbao J. (2002). Study of operating variables in the transformation of aqueous ethanol into hydrocarbons on an HZSM-5 zeolite. J. Chem. Technol. Biotechnol..

[28] Gayubo A.G., Alonso A., Valle B., Aguayo A.T., Bilbao J. (2010). Kinetic Model for the Transformation of Bioethanol into Olefins over a HZSM-5 Zeolite Treated with Alkali. Ind. Eng. Chem. Res..

[29] Gayubo A.G., Alonso A., Valle B., Aguayo A.T., Olazar M., Bilbao J. (2011). Kinetic modelling for the transformation of bioethanol into olefins on a hydrothermally stable Ni-HZSM-5 catalyst considering the deactivation by coke. Chem. Eng. J..

[30] Takahara I., Saito M., Inaba M., Murata K. (2005). Dehydration of Ethanol into Ethylene over Solid Acid Catalysts. Catal. Lett..

[31] Takahara I., Saito M., Matsuhasi H., Inaba M., Murata K. (2007). Increase in the number of acid sites of a H-ZSM 5 zeolite during the dehydration of ethanol. Catal. Lett..

[32] Inaba M., Murata K., Saito M., Takahara I. (2007). Production of olefins from ethanol by Fe-supported zeolite catalysts. Green Chem..

[33] Song Z., Takahashi A., Mimura N., Fujitani T. (2009). Production of Propylene from Ethanol Over ZSM-5 Zeolites. Catal. Lett..

[34] Song Z., Takahashi A., Nakamura I., Fujitani T. (2010). Phosphorus-modified ZSM-5 for conversion of ethanol to propylene. Appl. Catal. A Gen..

[35] Arias D., Colmenares A., Cubeiro M.L., Goldwasser J., Lopez C.M., Machado F.J., Sazo V. (1997). The transformation of ethanol over AlPO4 and SAPO molecular sieves with AEL and AFI topology. Kinetic and thermodynamic approach. Catal. Lett..

[36] Golay S., Doepper R., Renken A. (1999). Reactor performance enhancement under periodic operation for the ethanol dehydration over γ-alumina, a reaction with a stop-effect. Chem. Eng. Sci..

[37] Bakoyannakis D.N., Zamboulis D., Stalidis G.A., Deliyanni E.A. (2001). The effect of preparation method on the catalytic activity of amorphous aluminas in ethanol dehydration. J. Chem. Technol. Biotechnol..

[38] Doheim M.M., El-Shobaky H.G. (2002). Catalytic conversion of ethanol and iso-propanol over ZnO-treated Co_3_O_4_/Al_2_O_3_ solids. Colloids Surf. A.

[39] Zaki T. (2005). Catalytic dehydration of ethanol using transition metal oxide catalysts. J. Colloid Interface Sci..

[40] Varisli D., Dogu T., Dogu G. (2007). Ethylene and diethyl-ether production by dehydration reaction of ethanol over different heteropolyacid catalysts. Chem. Eng. Sci..

[41] Varisli D., Dogu T., Dogu G. (2008). Silicotungstic Acid Impregnated MCM-41-like Mesoporous Solid Acid Catalysts for Dehydration of Ethanol. Ind. Eng. Chem. Res..

[42] Varisli D., Dogu T., Dogu G. (2009). Novel Mesoporous Nanocomposite WOx-Silicate Acidic Catalysts: Ethylene and Diethylether from Ethanol. Ind. Eng. Chem. Res..

[43] Carrasco-Marn F., Mueden A., Moreno-Castilla C. (1998). Surface-Treated Activated Carbons as Catalysts for the Dehydration and Dehydrogenation Reactions of Ethanol. J. Phys. Chem. B.

[44] Kamiguchi S., Chihara T. (2003). Catalytic Dehydration of Alcohol to Olefin and Ether by Halide Clusters of Nb, Mo, Ta and W Possessing an Octahedral Metal Core. Catal. Lett..

[45] Kamiguchi S., Nagashima S., Komori K., Kodomari M., Chihara T. (2007). Thermal Activation of Molecular Tungsten Halide Clusters with the Retention of an Octahedral Metal Framework and the Catalytic Dehydration of Alcohols to Olefins as a Solid Acid Catalyst. J. Cluster Sci..

[46] Kamimura Y., Sato S., Takahashi R., Sodesawa T., Akashi T. (2003). Synthesis of 3-pentanone from 1-propanol over CeO_2_-Fe_2_O_3_ catalysts. Appl. Catal. A Gen..

[47] Nagashima O., Sato S., Takahashi R., Sodesawa T. (2005). Ketonization of carboxylic acids over CeO_2_-based composite oxides. J. Mol. Catal. A Chem..

[48] Tsuchida T., Kubo J., Yoshioka T., Sakuma S., Takeguchi T., Ueda W. (2008). Reaction of ethanol over hydroxyapatite affected by Ca/P ratio of catalyst. J. Catal..

[49] Iwamoto M., Kosugi Y. (2007). Highly Selective Conversion of Ethene to Propene and Butenes on Nickel Ion-Loaded Mesoporous Silica Catalysts. J. Phys. Chem. C.

[50] Ikeda K., Kawamura Y., Yamamoto T., Iwamoto M. (2008). Effectiveness of the template-ion exchange method for appearance of catalytic activity of Ni-MCM-41 for the ethene to propene reaction. Catal. Commun..

[51] Kasai K., Haishi T., Iwamoto M. (2007). Selective conversion of bio-ethanol to lower olefins on nickel ion-loaded mesoporous silica catalysts. Shokubai.

[52] Iwamoto M., Kasai K. Preparation of olefins from alcohols by use of ordered mesoporous catalysts in high yield. Jpn. Tokkyo Koho.

[53] Haishi T., Kasai K., Iwamoto M. (2011). Fast and Quantitative Dehydration of Lower Alcohols to Corresponding Olefins on Mesoporous Silica Catalyst. Chem. Lett..

[54] Iwamoto M., Kasai K., Haishi T. (2011). Conversion of Ethanol into Polyolefin Building Blocks: Reaction Pathways on Nickel Ion-loaded Mesoporous Silica. ChemSusChem.

[55] Sugiyama S., Kato Y., Wada T., Ogawa S., Nakagawa K., Sotowa K. (2010). Ethanol Conversion on MCM-41 and FSM-16, and on Ni-Doped MCM-41 and FSM-16 Prepared without Hydrothermal Conditions. Top. Catal..

[56] Liu B., Nakatani H., Terano M. (2003). Mechanistic implications of the unprecedented transformations of ethene into propene and butene over Phillips CrOx/SiO2 catalyst during induction period. J. Mol. Catal. A.

[57] Negishi E., Takahashi T. (1994). Patterns of Stoichiometric and Catalytic Reactions of Organozirconium and Related Complexes of Synthetic Interest. Acc. Chem. Res..

[58] Grubbs R.H. (2003). Handbook of Metathesis.

[59] Grubbs R.H. (2004). Olefin metathesis. Tetrahedron.

[60] Sato Y., Saito N., Mori M. (2002). Asymmetric Cyclization of ω-Formyl-1,3-dienes Catalyzed by a Zerovalent Nickel Complex in the Presence of Silanes. J. Org. Chem..

[61] Baker M.V., Brown D.H., Skelton B.W., White A.H. (2002). An investigation into alkenyl-functionalized 1,4,7-triazacyclononanes: Synthesis, metal complexation, and attempted olefin metathesis. Aust. J. Chem..

[62] Xiao S., Meng Z. (1994). X-ray photoelectron spectroscopy characterization of the reduction and oxidation behavior of Ni-containing HZSM-5 zeolites. J. Chem. Soc. Faraday Trans..

[63] Schoonheydt R.A., Roodhooft D. (1986). Spectroscopy of the thermal reduction of nickel(II) in the presence of hydrogen in zeolites X and Y. J. Phys. Chem..

[64] Elev I.V., Shelimov B.N., Kazanskii V.B. (1984). The role of nickel(1+) ions in the activity of NiCaY zeolite catalysts for ethylene dimerization. J. Catal..

[65] Prakash A.M., Wasowicz T., Kevan L. (1996). Reducibility, Location, and Adsorbate Interactions of Ni(I) Ions in Ni(II)-Exchanged Silicoaluminophosphate Type 41 Studied by Electron Spin Resonance and Electron Spin Echo Modulation Spectroscopies. J. Phys. Chem..

[66] Hartmann M., Poppl A., Kevan L. (1995). Formation and Stability of Ni(I) Ions in MCM-41 Mesoporous Molecular Sieves. J. Phys. Chem..

[67] Taoufik M., Le Roux E., Thivolle-Cazat J., Basset J.M. (2007). Direct transformation of ethylene into propylene catalyzed by a tungsten hydride supported on alumina: trifunctional single-site catalysis. Angew. Chem. Int. Ed..

[68] Lin B., Zhang Q., Wang Y. (2009). Catalytic Conversion of Ethylene to Propylene and Butenes over H-ZSM-5. Ind. Eng. Chem. Res..

[69] Tsuji K., Uchida H., Nakajou T., Sano K. (2008). Development of Novel Production Processes for Ethyl Acetate and Acetic Acid Catalyzed by Solid Heteropolyacids. Chem. Chem. Ind..

[70] Clause O., Kermarec M., Bonneviot L., Villain F., Che M. (1992). Nickel(II) ion-support interactions as a function of preparation method of silica-supported nickel materials. J. Am. Chem. Soc..

[71] Carriat J.Y., Che M., Kermarec M., Verdaguer M., Michalowicz A. (1998). Control of Dispersion of Ni^2+^ Ions via Chelate Ligands in the Preparation of Ni/SiO_2_ Materials. A XAFS Study. J. Am. Chem. Soc..

[72] Yang Y., Lim S., Du G., Chen Y., Ciuparu D., Haller G.L. (2005). Synthesis and Characterization of Highly Ordered Ni-MCM-41 Mesoporous Molecular Sieves. J. Phys. Chem. B.

[73] Martin G.A., Renoupre A., Dalmaiim G., Imelik B. (1970). Synthesis of nickel talc and antigorite. Study of their thermal decomposition and reduction to obtain nickel catalysts on silica. J. Chim. Phys..

[74] Wells A.F. (1984). Structural Inorganic Chemistry.

[75] Farges F., Munoz M., Siewert R., Malavergne V., Brown G.E., Behrens H., Nowak M., Petit P.E. (2001). Transition elements in water-bearing silicate glasses/melts. Part II. Ni in water-bearing glasses. Geochim. Cosmochim. Acta.

[76] Burattin P., Che M., Louis C. (1997). Characterization of the Ni(II) Phase Formed on Silica Upon Deposition-Precipitation. J. Phys. Chem. B.

[77] Yang J.C., Shul Y.G., Louis C., Che M. (1998). In situ EXAFS study of the nucleation and crystal growth of Ni particles on SiO_2_ support. Catal. Today.

[78] Bonneviot L., Clause O., Che M., Manceau A., Decarreau A., Villian F., Bazin D., Dexpert H. (1989). Investigation by EXAFS of the effect of pH on the structure of nickel(2+) ions impregnated on silica. Phys. B.

[79] Clause O., Bonneviot L., Che M., Dexpert H. (1991). EXAFS characterization of the adsorbed state of nickel(II) ions in nickel/silica materials prepared by deposition-precipitation. J. Catal..

[80] Espinos J.P., Gonzalez-Elipe A.R., Munuera G., Garcia J., Conesa J.C., Burattini E. (1989). EXAFS study of catalyst preparation procedure in nickel-silica and nickel-titania. Phys. B.

[81] Grauby O., Petit S., Decarreau A., Baronenet A. (1993). The beidellite-saponite series: An experimental approach. Eur. J. Mineral..

[82] Decarreau A. (1985). Partitioning of divalent transition elements between octahedral sheets of trioctahedral smectites and water. Geochim. Cosmochim. Acta.

[83] Hadjiivanov K., Mihaylov M., Klissurski D., Stefanov P., Abadjieva N., Vassileva E., Mintchev L. (1999). Characterization of Ni/SiO_2_ Catalysts Prepared by Successive Deposition and Reduction of Ni^2+^ Ions. J. Catal..

[84] Wojcieszak R., Moteverdi S., Mercy M., Nowak I., Ziolek M., Bettahar M.M. (2004). Nickel containing MCM-41 and AlMCM-41 mesoporous molecular sieves. Characteristics and activity in the hydrogenation of benzene. Appl. Catal. A Gen..

[85] Kutseva L.N. (1961). Influence of superstoichiometric oxygen on the catalytic, adsorptive, and electrical properties of nickelous oxide. Dokl. Akad. Nauk. SSSR.

[86] Houalla M., Delannay F., Matsuura I., Delmon B. (1980). Physico-chemical characterization of impregnated and ion-exchanged silica-supported nickel oxide. J. Chem. Soc. Faraday. Trans. 1.

[87] Kermarec M., Carriat J.Y., Burattin P., Che M., Decarreau A. (1994). FTIR Identification of the Supported Phases Produced in the Preparation of Silica-Supported Nickel Catalysts. J. Phys. Chem..

[88] Lehmann T., Wolff T., Zahn V.M., Veit P., Hamel C., Seidel-Morgenstern A. (2011). Preparation of Ni-MCM-41 by equilibrium adsorption—Catalytic evaluation for the direct conversion of ethene to propene. Catal. Commun..

[89] Xu S., Wang X. (2005). Highly active and coking resistant Ni/CeO_2_-ZrO_2_ catalyst for partial oxidation of methane. Fuel.

[90] Li G., Hu L., Hill J.M. (2006). Comparison of reducibility and stability of alumina-supported Ni catalysts prepared by impregnation and co-precipitation. Appl. Catal. A.

[91] Abe T., Tachibana Y., Uematsu T., Iwamoto M. (1995). Preparation and characterization of Fe2O3 nanoparticles in mesoporous silicate. J. Chem. Soc. Chem. Commun..

[92] Iwamoto M., Tanaka Y. (2001). Preparation of metal ion-planted mesoporous silica by template ion-exchange method and its catalytic activity for asymmetric oxidation of sulfide. Catal. Surv. Jpn..

[93] Hayashi F., Iwamoto M. (2010). Effect of pore structure on the nitridation of mesoporous silica with ammonia. Eur. J. Inorg. Chem..

